# Innovative regression model-based decision support tool for optimizing radiotherapy techniques in thoracic esophageal cancer

**DOI:** 10.3389/fonc.2024.1370293

**Published:** 2024-07-24

**Authors:** Yuxing Li, Yue Ke, Xinran Huang, Ruijuan Zhang, Wanghui Su, Hongbing Ma, Pu He, Xinyue Cui, Shan Huang

**Affiliations:** Department of Radiation Oncology, the Second Affiliated Hospital of Xi’an Jiaotong University, Xi’an, China

**Keywords:** thoracic esophageal cancer, radiotherapy, VMAT, IMRT, decision support tool

## Abstract

**Background:**

Modern radiotherapy exemplified by intensity-modulated radiation therapy (IMRT) and volumetric modulated arc therapy (VMAT), has transformed esophageal cancer treatment. Facing challenges in treating thoracic esophageal cancer near vital organs, this study introduces a regression model-based decision support tool for the optimal selection of radiotherapy techniques.

**Methods:**

We enrolled 106 patients diagnosed with locally advanced thoracic esophageal cancer in this study and designed individualized IMRT and VMAT radiotherapy plans for each patient. Detailed dosimetric analysis was performed to evaluate the differences in dose distribution between the two radiotherapy techniques across various thoracic regions. Single-factor and multifactorial logistic regression analyses were employed to establish predictive models (P1 and P2) and factors such as TLV/PTV ratio. These models were used to predict the compliance and potential advantages of IMRT and VMAT plans. External validation was performed in a validation group of 30 patients.

**Results:**

Using predictive models, we developed a data-driven decision support tool. For upper thoracic cases, VMAT plans were recommended; for middle/lower thoracic cases, the tool guided VMAT/IMRT choices based on TLV/PTV ratio. Models P1 and P2 assessed IMRT and VMAT compliance. In validation, the tool showed high specificity (90.91%) and sensitivity (78.95%), differentiating IMRT and VMAT plans. Balanced performance in compliance assessment demonstrated tool reliability.

**Conclusion:**

In summary, our regression model-based decision support tool provides practical guidance for selecting optimal radiotherapy techniques for thoracic esophageal cancer patients. Despite a limited sample size, the tool demonstrates potential clinical benefits, alleviating manual planning burdens and ensuring precise, individualized treatment decisions for patients.

## 1 Introduction

Esophageal cancer, a prevalent malignancy globally, has consistently ranked among the top ten causes of cancer-related deaths (1, 2). Radiotherapy remains a primary therapeutic modality for esophageal cancer, particularly in the treatment of locally advanced and advanced stages (3, 4). However, the cure rate for radiotherapy in esophageal cancer is below 15% (5). Local uncontrolled growth and recurrence constitute the primary reasons for the failure of radiotherapy in esophageal cancer (6).

The evolution of modern radiotherapy techniques, notably intensity-modulated radiation therapy (IMRT) and volumetric modulated arc therapy (VMAT), has ushered in a new era of possibilities for treating esophageal cancer (7, 8). VMAT is a form of IMRT that delivers radiation by rotating the linear accelerator around the patient. This technique allows for a more precise and targeted approach, reducing exposure to surrounding healthy tissue and critical organs. The introduction and optimization of VMAT have significantly enhanced the effectiveness and efficiency of radiation therapy in cancer treatment, providing better outcomes and fewer side effects compared to conventional methods (9–11). However, treating thoracic esophageal cancer, situated proximally to critical organs such as the heart and lungs, demands a meticulous balance between fulfilling target dose requirements and minimizing radiation exposure (12, 13).

Studies reveal nuanced advantages of IMRT and VMAT in treating thoracic esophageal cancer, showcasing the intricate decision-making landscape (14, 15). Early initiation of radiotherapy emerges as a pivotal factor in enhancing survival rates for patients with thoracic esophageal cancer (4, 5). However, the challenging task of determining which patients stand to benefit the most from VMAT, especially in cases with limited VMAT advantages, highlights the pressing need for a decision support tool (13, 16).

This study is dedicated to comparing the advantages of IMRT and VMAT, identifying key factors influencing the choice between these techniques for thoracic esophageal cancer, and developing a clinical decision support tool. This tool not only proactively assesses plan compliance but also recommends adjustments for non-compliant plans. By minimizing treatment delays and optimizing resource use, it empowers physicians in informed decision-making based on reliable benefit estimates, with a particular focus on the pivotal role of radiotherapy in the overall treatment paradigm.

## 2 Materials and methods

### 2.1 Patient cohort

A retrospective analysis was conducted on 136 patients diagnosed with thoracic esophageal squamous cell carcinoma at the Department of Radiation Oncology, the Second Affiliated Hospital of Xi’an Jiaotong University, from February 2016 to December 2019. Inclusion criteria encompassed pathological diagnosis of esophageal squamous cell carcinoma, American Joint Committee on Cancer (AJCC) stage II-III, and a primary tumor located in the thoracic esophagus, deemed unresectable by surgical evaluation or refusal of surgical intervention. Exclusion criteria comprised a history of previous radiotherapy, chemotherapy, immunotherapy, or biologic therapy, concurrent infection, symptoms of esophageal perforation, significant esophageal ulceration, bleeding risk, and severe cardiopulmonary diseases.

### 2.2 Patient immobilization and CT simulation localization

Patients were immobilized in the supine position using a thermoplastic body mold. CT simulation and localization scans were performed using the Siemens AS 20 CT simulator, with optional contrast-enhanced scans using iodized oil for intravenous contrast. The slice thickness was 0.5 cm. Laser lights were used as reference points on the body mold, and the scanning range extended from the mandible to the adrenal glands. CT images were transferred to the Eclipse 13.6 three-dimensional treatment planning system.

### 2.3 Radiation target delineation

According to the guidelines set forth in the International Commission on Radiation Units and Measurements (ICRU) Report 83 (17), our methodology for target delineation adhered to rigorous standards and involved meticulous naming and delineation processes, validated by two experienced attending physicians. Initially, we defined the gross tumor volume (GTV), comprising the primary tumor lesion (GTV-T) and regional metastatic lymph nodes (GTV-N), detected through a comprehensive array of imaging modalities, including endoscopy, barium swallow, endoscopic ultrasound, CT, and PET/CT. Lymph nodes were identified based on a short-axis diameter ≥1 cm on CT and/or MR, with specific considerations for periesophageal and tracheoesophageal grooves. Subsequently, the clinical target volume (CTV) was determined, extending typically from the GTV to cover potential residual tumor and lymphatic drainage regions. CTV delineation involved selective irradiation of lymphatic drainage areas with appropriate expansions superior-inferiorly, anterior-posteriorly, and laterally. Notably, in the upper thoracic segment, CTV encompassed bilateral lymph node drainage regions (levels 101, 104, 105, 106, 107, and 108), while in the middle thoracic segment, it included levels 105, 106, 107, 108, and partially 110, and abdominal levels 1, 2, and 3 lymph node drainage regions. For the lower thoracic segment, it encompassed levels 107, 108, and 110, and abdominal levels 1, 2, and 3 drainage regions. Attention was given to avoiding adjacent anatomical barriers during target delineation. Finally, we defined the planning target volume (PTV) and planning gross tumor volume (PGTV) to ensure optimal radiation dose coverage while minimizing exposure to surrounding critical organs. Specialized radiation oncologists meticulously contoured organs at risk (OARs), including the left lung, right lung, spinal cord, heart, stomach, and liver, with adjustments made to window width and level to ensure contouring accuracy and completeness.

### 2.4 Treatment planning

The treatment planning process is meticulously executed using the Eclipse™ treatment planning system version 13.6 to finely tune the distribution of radiation dose. Both IMRT and VMAT entail a meticulous approach, involving the precise selection of beam angles, modulation of beam intensity, and accurate calculation of dose distributions to achieve optimal coverage of the PTV while minimizing exposure to OARs. Across all plans, the prescription dose is uniformly set at 25 fractions of 5000 cGy, with stringent adherence to ensuring that 95% of the PTV receives the prescribed dose. Furthermore, rigorous criteria are enforced to maintain minimum and maximum doses within the PTV within the range of 95% to 107% of the prescription dose, thereby mitigating the risk of underdosing or overdosing scenarios. Specialized attention is devoted to eliminating cold spots within the PTV and averting hot spots on the diseased esophageal wall to optimize treatment efficacy. Precise dose constraints are established for OARs, including maximum doses to the spinal cord (≤ 4500 cGy) and total lungs (V20 ≤ 25%, V5 ≤ 60%, Dmean ≤ 1300 cGy), as well as the heart (V30 ≤ 40%, V40 ≤ 30%, Dmean ≤ 2600 cGy). Each patient’s treatment plan is meticulously executed under the close supervision of seasoned radiation therapists, in collaboration with senior physicists, to ensure strict adherence to quality assurance protocols and to optimize treatment outcomes. The treatment plans are developed utilizing the Varian Eclipse 13.6 treatment planning system and the Varian Trilogy linear accelerator equipped with 6MV X-rays. For IMRT, a 5-field non-uniform distribution irradiation plan is employed, while VMAT utilizes a 2-arc design with sector avoidance strategies. The optimization of IMRT and VMAT is guided by the prescription dose and OAR constraints, with adjustments made to weighting and fine-tuning of dose constraints to achieve an optimal dose distribution.

### 2.5 Plan evaluation

The radiotherapy plans were evaluated based on dose-volume histograms (DVH) and referenced the ICRU Report 83 (17), NCCN Clinical Practice Guidelines in Oncology (18), and Quantitative Analyses of Normal Tissue Effects in the Clinic (QUANTEC) (19). Considering the study’s focus on patients with esophageal squamous cell carcinoma, and China’s status as a major country for esophageal squamous cell carcinoma incidence, we also consulted the Chinese guidelines for esophageal cancer radiotherapy (20, 21). Specific criteria included ensuring that the prescription dose curve covered 95% of the PTV, with the 95% dose curve corresponding to 99% of the PTV. The maximum allowable dose within the PTV was set at 110% of the prescription dose. Furthermore, unevenness correction was performed. Conformity index (CI) and homogeneity index (HI) were used to assess the conformity and homogeneity of the target volume (22, 23). A CI value between 0 and 1 indicates better conformity as it approaches 1, while a lower HI value indicates better dose distribution uniformity. Strict dose constraints were set for OARs, including: total lung Dmean ≤ 1300 cGy, V20 ≤ 25%, V30 ≤ 20%, V5 ≤ 60%; heart Dmean ≤ 2600 cGy, V30 ≤ 40%, V40 ≤ 30%; and maximum spinal cord dose ≤ 4500 cGy. Plans were considered compliant only if all parameters met the dose constraint values. Compliant plans were further assessed using optimization criteria, particularly favoring lower heart doses when there were no significant differences in lung doses between two plans.

### 2.6 Development of predictive models P1 and P2

During the model construction phase, we conducted a rigorous statistical analysis to develop two predictive models, designated as P1 and P2, tailored for intensity-modulated radiation therapy (IMRT) plans and volumetric modulated arc therapy (VMAT) plans, respectively. These models aimed to predict the likelihood of treatment plan compliance based on a set of carefully selected measurement parameters.

For both P1 and P2 models, we initially performed single-factor logistic analysis to assess the correlation between individual measurement parameters and plan compliance, considering a significance level of p< 0.1. Subsequently, we employed a multi-factor logistic stepwise regression approach to identify the most influential variables to include in the regression equations.

In the multi-factor logistic regression analysis, the coefficients for each variable were calculated using an iterative process that aims to minimize the model’s deviance. This process involves sequentially adding and removing variables based on their contribution to the model’s goodness-of-fit, as assessed by statistical metrics such as the Akaike information criterion (AIC) or the likelihood ratio test (LRT). The final set of coefficients represents the estimated impact of each predictor variable on the log-odds of plan compliance.

The variables selected for the P1 model, intended for IMRT plans, were PTV volume and TLV/PTV ratio. The resulting regression equation for the P1 model is formulated as follows:


(1) $$P1=\frac{1}{1+e^{-\left(-0.816-0.513\frac{TLV}{PTV}+0.024\cdot PTV\;volume\right)}}$$

For the P2 model, designed for VMAT plans, the key predictors were TLV/PTV ratio and PTV length. The regression equation for the P2 model is represented as:


(2) $$P2=\frac{1}{1+e^{-\left(-1.442-0.522\frac{TLV}{PTV}+0.641\cdot PTV\;length\right)}}$$

To validate the stability of both models, we employed the Hosmer and Lemeshow Test, which confirmed their reliability. Furthermore, the predictive performance of the models was assessed using receiver operating characteristic (ROC) curve analysis, demonstrating their effectiveness in predicting treatment plan compliance. To address overfitting in developing predictive models P1 and P2, we employed stringent feature selection criteria and conducted both univariate and multivariate logistic regression analyses. Rigorous model validation techniques, including the Hosmer and Lemeshow test and ROC curve analysis, were utilized to ensure predictive performance. Additionally, external validation with an independent dataset of 30 patients confirmed the models’ generalizability and robustness. The measurement parameters utilized in the models were defined as follows: planning target volume (PTV), total lung volume (TLV), the ratio of TLV to PTV (TLV/PTV), the ratio of total heart volume to PTV (THV/PTV), and PTV length. These parameters were directly measured or delineated using the Varian Eclipse Treatment Planning System (TPS). The regression model-based decision support tool has been made publicly available at GitHub repository (https://github.com/Huangshan2164/IMRT_VMAT_Decision_Support_Tool).

### 2.7 Statistical analysis

Shapiro-Wilk test was used for normality testing of dosimetric parameters, including PTV D2, D98, D50, D95, CI, HI, heart V30, V40 and Dmean, lung V5, V10, V20, Dmean, spinal cord Dmax, and monitor units (MU), among others. For parameters that met normal distribution, paired t-tests were used, and for those not meeting normal distribution, Wilcoxon tests were applied. Single-factor logistic analysis was used to screen influencing factors, and multi-factor logistic binary regression was employed to establish predictive models. The stability of the models was assessed using the Hosmer and Lemeshow Test. Critical values were determined using ROC curves, and the applicability of related parameters for predicting plans was evaluated. Statistical analysis was performed using SPSS 18.0 software, with p< 0.05.

## 3 Results

### 3.1 Patients’ characteristics of training group

**Figure 1:**
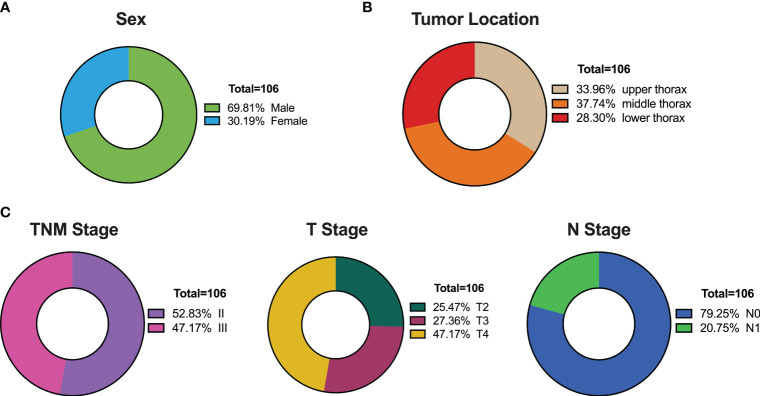
Patient Characteristics in the Training Set of 106 Thoracic Esophageal Cancer Cases. **(A)** Gender distribution. **(B)** Distribution of tumor locations. **(C)** Distribution of tumor staging.

This investigation comprised 106 patients diagnosed with thoracic esophageal cancer, with a median age of 68 years and a notable male predominance (constituting 2/3 of the cohort), consistent with the epidemiology of esophageal cancer (**Figure 1A**). Tumor characteristics revealed a median length of 6.75 cm, with distribution across the upper (36 cases, 33.96%), middle (40 cases, 37.74%), and lower (30 cases, 28.30%) thoracic regions (**Figure 1B**). Staging demonstrated an even distribution between Stage II and Stage III, with T4 stage being the predominant stage (47.17%), and N0 accounting for 79.25% of the cases (**Figure 1C**).

### 3.2 Dose-volumetric comparison

**Figure 2:**
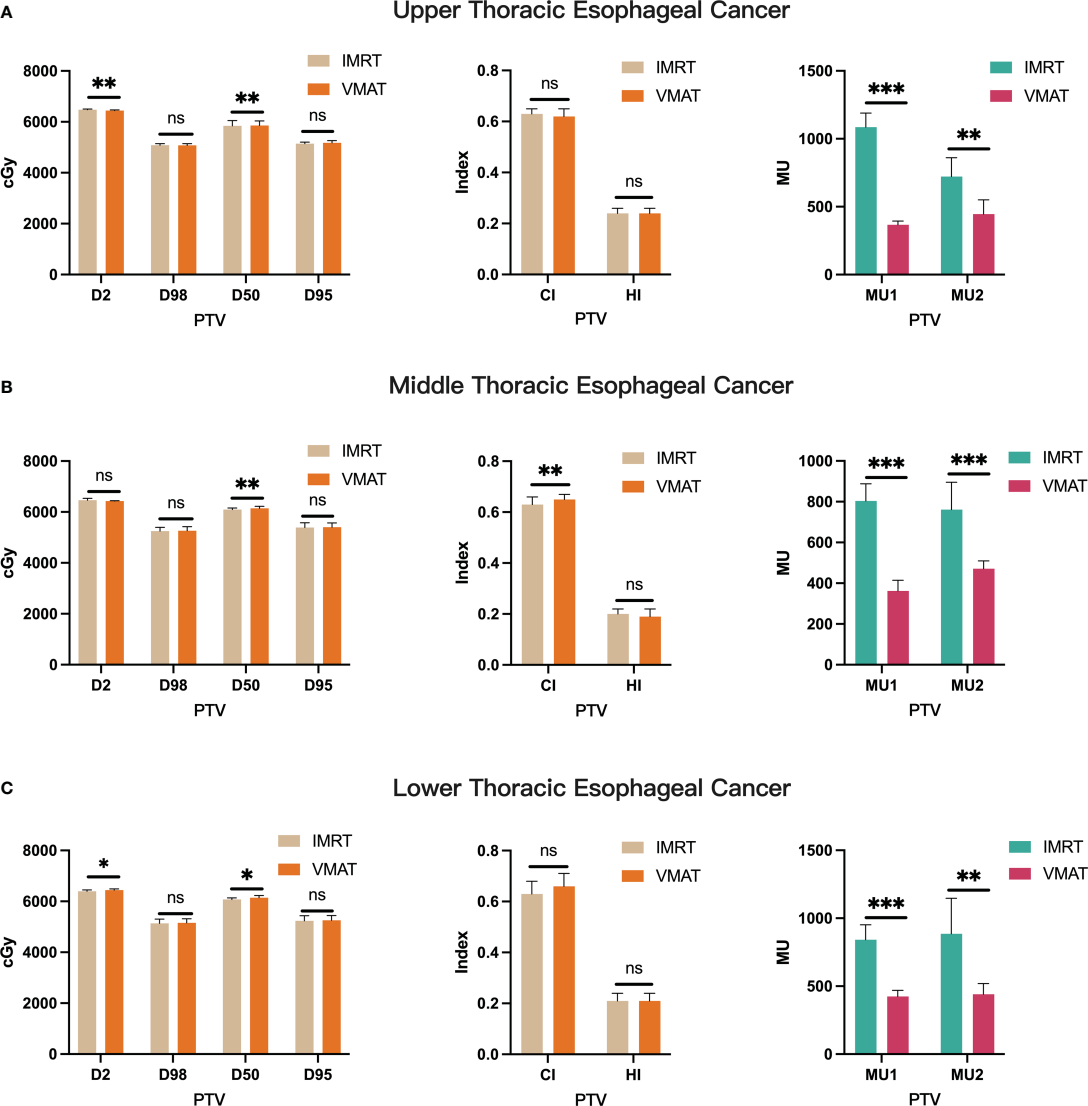
Comparative Analysis of PTV Parameters between IMRT and VMAT for 106 Thoracic Esophageal Cancer Patients. Comparison in the upper thoracic **(A)**, middle thoracic **(B)**, and lower thoracic **(C)** regions. PTV, planning target volume; MU, monitor units; CI, conformity index; HI, homogeneity index. *p< 0.05; **p< 0.01; ***p< 0.001; ns, non-significant.

In our study, we meticulously crafted individualized IMRT and VMAT plans for each of the 106 patients diagnosed with thoracic esophageal cancer. Focusing on upper thoracic cancer (**Figure 2A**), our analysis revealed that both VMAT and IMRT plans achieved similar PTV coverage (D98, D95, p > 0.05), yet VMAT exhibited a notable reduction in MU (p< 0.05), indicating enhanced treatment efficiency. Transitioning to the middle thoracic region (**Figure 2B**), while VMAT matched IMRT in PTV coverage (D98, D95, p > 0.05), it showcased superior conformity (CI, p< 0.05) and required fewer MU (p< 0.05), underscoring its precision and resource optimization benefits. Similarly, in cases of lower thoracic cancer (**Figure 2C**), VMAT plans maintained comparable PTV coverage (D98, D95, p > 0.05) while significantly reducing MU (p< 0.05), thereby optimizing dose distribution and potentially reducing complications. Through a thorough investigation across upper, middle, and lower thoracic regions, illustrated in **Supplementary Figure S1**, we meticulously evaluated the dose distribution for the PTV and PGTV, providing comprehensive insights via DVH. Overall, our findings elucidate the consistent advantage of VMAT over IMRT in treatment efficiency, highlighting its potential to minimize treatment-related side effects and optimize clinical resource utilization.

### 3.3 Dose-volumetric comparison of organs at risk

**Figure 3:**
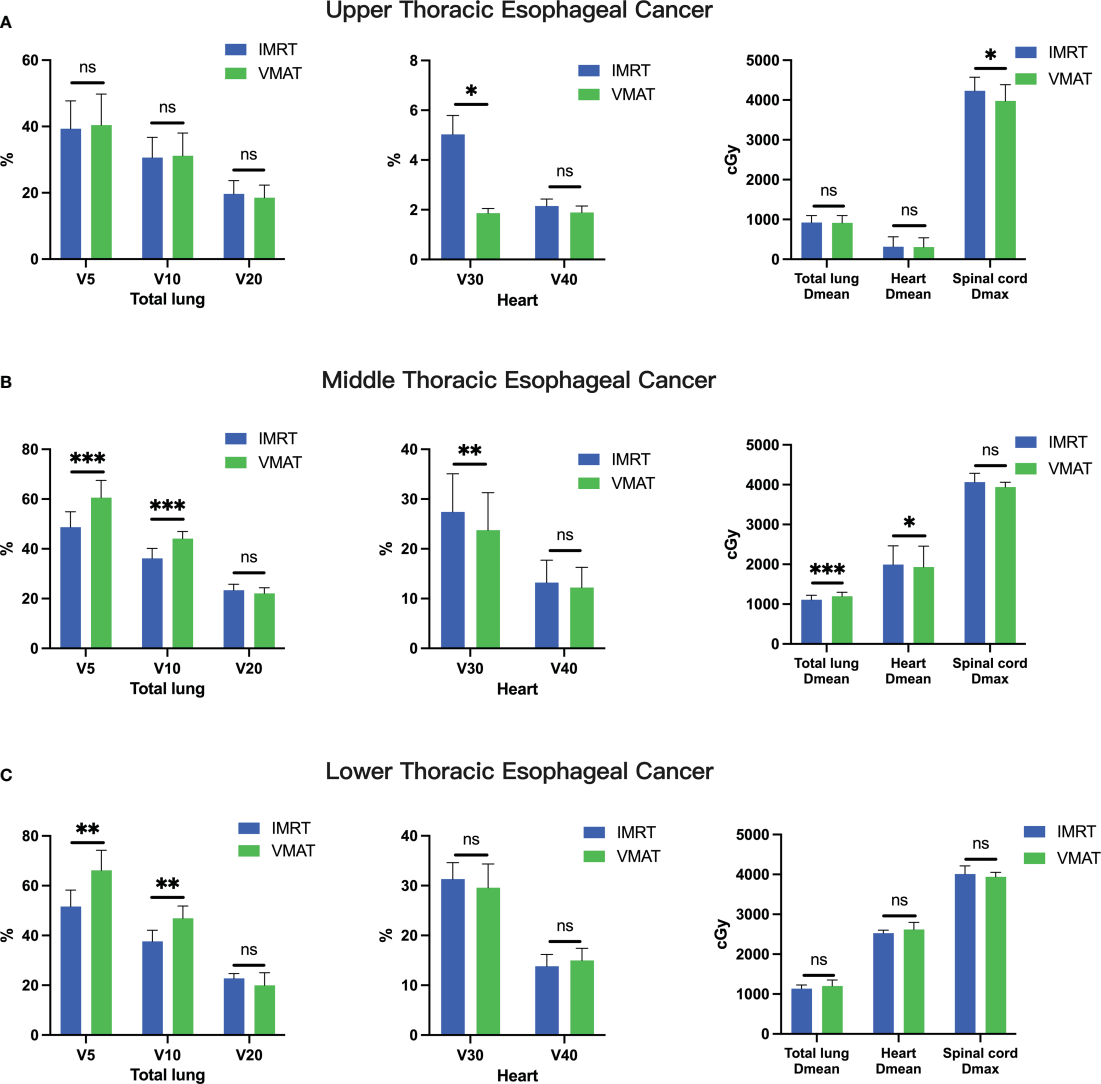
Comparative Analysis of OAR Parameters between IMRT and VMAT for 106 Thoracic Esophageal Cancer Patients. Comparison in the upper thoracic **(A)**, middle thoracic **(B)**, and lower thoracic **(C)** regions. OAR, organ at risk. *p< 0.05; **p< 0.01; ***p< 0.001; ns, non-significant.

Furthermore, a thorough comparison of dosimetric parameters for organs at risk, including the total lung, heart, and spinal cord, was conducted between VMAT and IMRT radiotherapy techniques. For upper thoracic esophageal cancer, VMAT and IMRT plans showed no significant difference in total lung dose (V5, V10, and V20; all p > 0.05). However, VMAT exhibited a notable reduction in heart V30 (p< 0.05) and spinal cord Dmax (p< 0.05, **Figure 3A**). In contrast, for middle and lower thoracic esophageal cancer, VMAT increased the total lung dose, with significantly higher V5 and V10 than IMRT plans (p< 0.05, **Figures 3B, C**). Nevertheless, VMAT plans demonstrated significantly lower heart V30 (p< 0.05) and heart Dmean (p< 0.05) for middle thoracic esophageal cancer (**Figure 3B**).

### 3.4 Distribution and preference of IMRT and VMAT plans across thoracic segments

**Figure 4:**
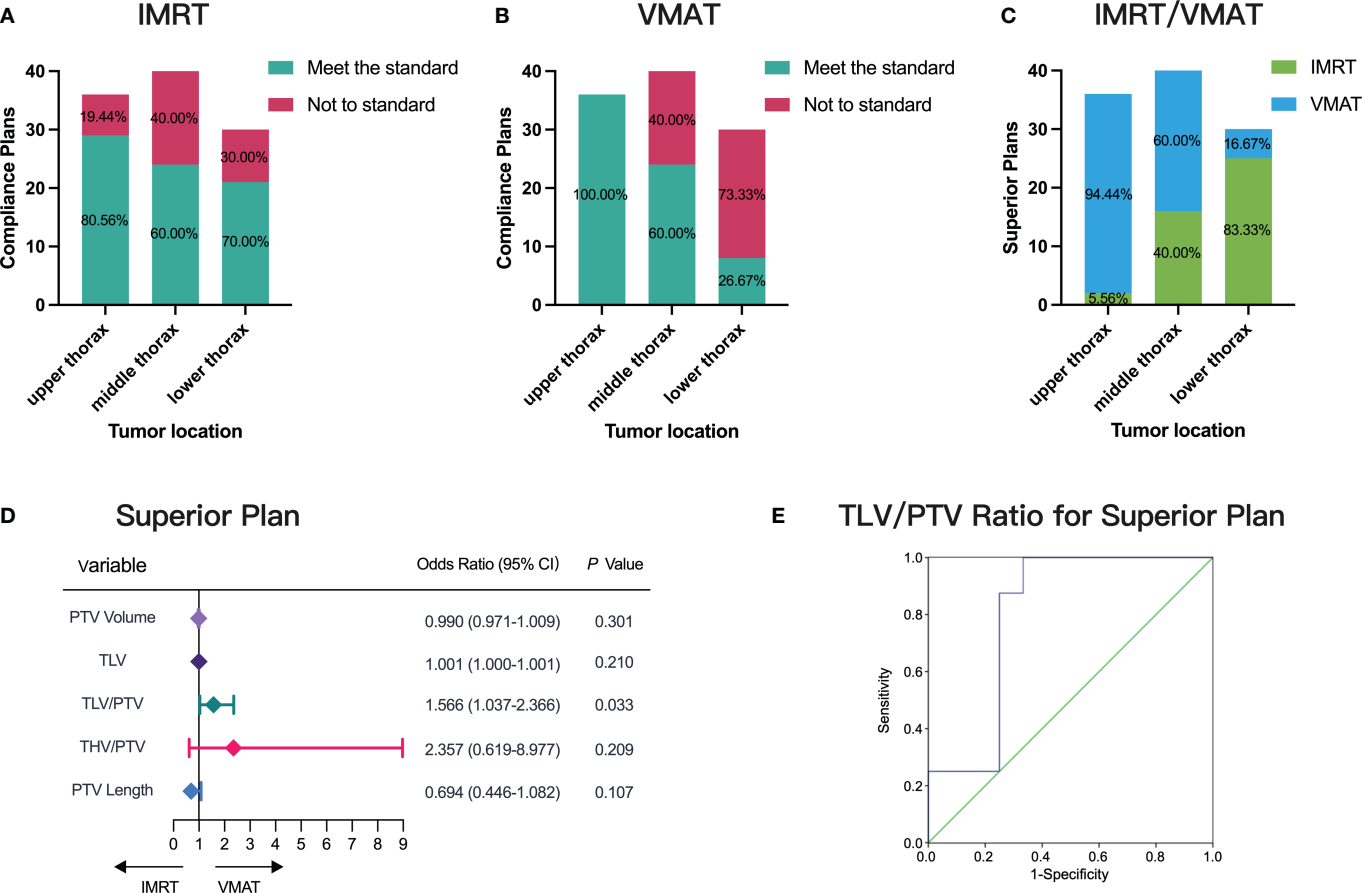
Distribution and Preference of IMRT and VMAT Plans Across Thoracic Segments. **(A)** Compliance assessment for IMRT and VMAT in different tumor locations. **(B)** Compliance assessment for VMAT in different tumor locations. **(C)** Evaluation of superior plans between IMRT and VMAT. **(D)** Single-factor logistic regression analysis correlating measurement parameters with superior plan selection. **(E)** ROC curve predicting superior plan selection based on TLV/PTV ratio, AUC= 0.802.

We assessed the distribution and preference of IMRT and VMAT plans among 106 patients across varying thoracic segments. Among the 36 upper thoracic patients, 29 demonstrated compliance with IMRT plans, while 36 adhered to VMAT plans, indicating a higher compliance rate with VMAT (**Figures 4A, B**). In the middle thoracic segment, comprising 40 patients, 24 patients adhered to both IMRT and VMAT plans, suggesting similar compliance rates between the two techniques (**Figures 4A, B**). However, in the lower thoracic segment with 30 patients, 21 patients adhered to IMRT plans, while only 8 adhered to VMAT plans, highlighting higher compliance with IMRT (**Figures 4A, B**).

Further analysis revealed superior plans between IMRT and VMAT (**Figure 4C**). Among the 36 upper thoracic patients, 34 preferred VMAT plans, accounting for 94.44%. In the middle thoracic segment with 40 patients, 24 preferred VMAT plans, representing 60% of the cohort. Conversely, in the lower thoracic segment with 30 patients, 25 preferred IMRT plans, comprising 83.33% of the group. These findings underscore VMAT’s superiority in upper thoracic esophageal cancer cases.

In addressing the uncertainty surrounding the choice between VMAT and IMRT for middle and lower thoracic cancer patients, our comprehensive analysis revealed a significant correlation between the TLV/PTV ratio and the likelihood of selecting superior plans (p< 0.05, **Figure 4D**). ROC curve analysis demonstrated the TLV/PTV ratio’s good predictive performance, with an AUC of 0.802 (95% CI, 0.599-1.000; **Figure 4E**). An optimal threshold of 12.59 exhibited 62.50% sensitivity and 75.00% specificity. Beyond this threshold, VMAT plans excelled in middle and lower thoracic cancer cases, highlighting the TLV/PTV ratio’s predictive value in guiding treatment decisions.

### 3.5 Establishment of compliance prediction models P1 and P2

**Figure 5:**
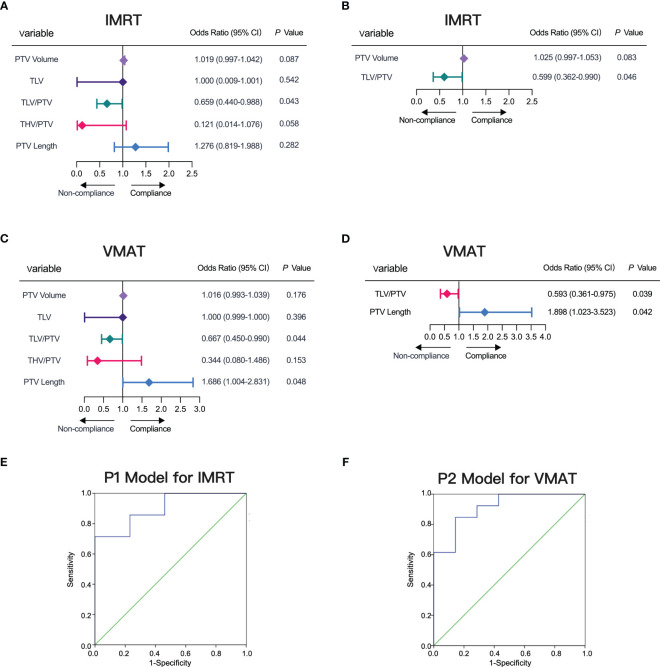
Factors Influencing IMRT and VMAT Plan Compliance. **(A, B)** Factors influencing IMRT plan compliance depicted in forest plots for single-factor and multi-factor logistic regression analyses. **(C, D)** Factors influencing VMAT plan compliance depicted in forest plots for single-factor and multi-factor logistic regression analyses. **(E)** ROC curve for model P1 predicting IMRT plan compliance, AUC= 0.901. **(F)** ROC curve for model P2 predicting VMAT plan compliance, AUC= 0.912.

We systematically evaluated factors influencing compliance in IMRT and VMAT plans, leading to the development of predictive models (P1 for IMRT (Equation 1) and P2 for VMAT (Equation 2)). Our analyses, including single-factor logistic analysis (**Figures 5A, C**) and multifactorial logistic regression (**Figures 5B, D**), highlighted the significance of TLV/PTV ratio and specific PTV characteristics (volume for IMRT and length for VMAT) in ensuring plan compliance.

To validate our models, we used P1 for IMRT and P2 for VMAT as test variables in ROC curve analysis. The ROC results demonstrated a high AUC of 0.901 (95% CI, 0.754-1.000) for P1, achieving a sensitivity of 71.43% and specificity of 84.62% at the optimal threshold of 0.72 (**Figure 5E**). Similarly, for P2, we observed an AUC of 0.912 (95% CI, 0.783-1.000), with a sensitivity of 84.62% and specificity of 85.71% at the threshold value of 0.62 (**Figure 5F**). Along with the detailed prediction score in **Supplementary Table S1**, our ROC curve analysis confirmed the robust predictive performance of P1 and P2, highlighting their effectiveness in predicting compliance for both IMRT and VMAT plans.

### 3.6 Development of the decision support tool

**Figure 6:**
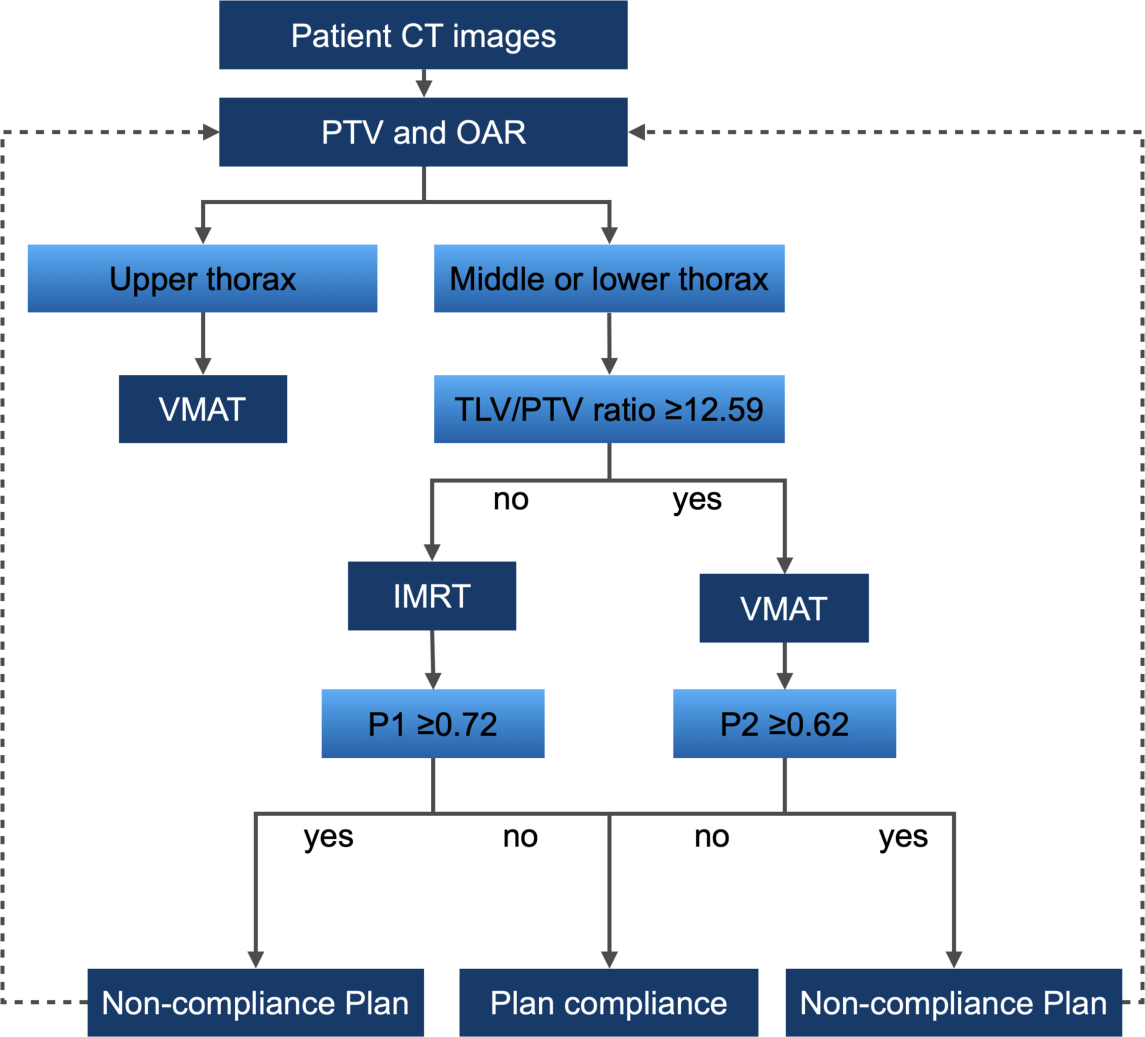
Decision Support Tool Workflow for Thoracic Esophageal Cancer Radiotherapy. Workflow based on models P1, P2, and the threshold of TLV/PTV ratio in the Training Group.

Building upon models P1 and P2 and utilizing the TLV/PTV ratio threshold, we developed a decision support tool for thoracic esophageal cancer radiotherapy (**Figure 6**). This involves CT simulation to acquire images suitable for treatment planning, followed by PTV and OAR delineation by medical professionals. The tool recommends VMAT plans for upper thoracic esophageal cancer patients and employs the TLV/PTV ratio for guidance in choosing between VMAT and IMRT plans for middle or lower thoracic esophageal cancer. Model P1 affirms its utility in predicting plan compliance for IMRT, while model P2 similarly affirms its utility in predicting plan compliance for VMAT. If the predictive probabilities of models P1 and P2 exceed predefined thresholds, it suggests the possibility of the plans not complying with the specified criteria. In such cases, adjustments to the target area are recommended, such as extending or reducing the treatment volume, modifying the beam angles, or altering the dose distribution within the target. Following these adjustments, a reassessment using the decision support tool is warranted.

### 3.7 External validation of the decision support tool

**Figure 7:**
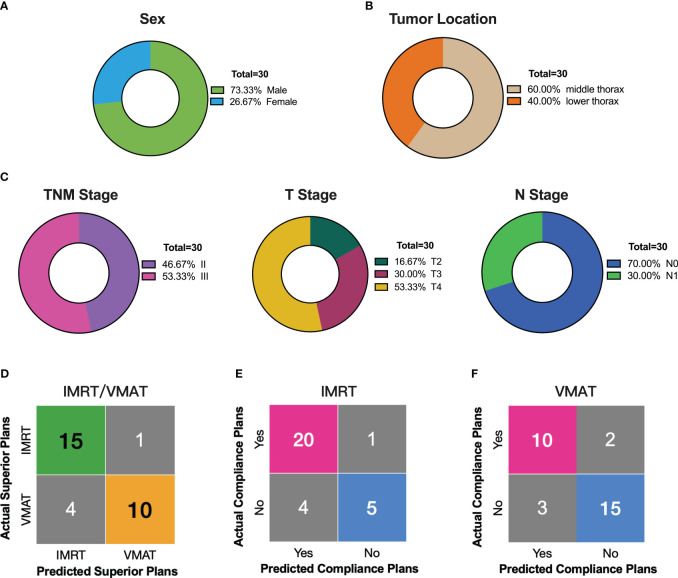
External Validation of the Decision Support Tool in 30 Thoracic Esophageal Cancer Patients. **(A–C)** Distribution of sex, tumor location, and stage in the external validation group of 30 patients. **(D)** Comparison of decision support tool predictions with actual outcomes for superior radiotherapy plans. **(E)** Comparing predicted compliant plans to actual IMRT compliance plans using the decision support tool. **(F)** Comparing predicted compliant plans to actual VMAT compliance plans using the decision support tool.

In a validation study with 30 external patients, we assessed our decision support tool’s reliability. The median age of the validation group was 71 years, mostly males (2/3 of participants, **Figure 7A**). Tumor lengths (median 6.30 cm) were distributed across 18 mid-chest and 12 lower chest cases (**Figure 7B**). Staging showed half in stage II and III, with T4 (53.33%) and N0 (70.00%) predominating (**Figure 7C**). Applying the decision support tool to predict optimal IMRT and VMAT plans for the 30 validation cases, we then compared the predictions against the actual plans. The results demonstrated a high level of specificity (90.91%) and sensitivity (78.95%) in distinguishing between IMRT and VMAT plans (**Figure 7D**). Furthermore, the tool was utilized to predict plan compliance for both IMRT and VMAT, and these predictions were compared with the actual outcomes (**Figures 7E, F**). The predictive workflow exhibited notable sensitivity (95.24%) for IMRT plan compliance. Concerning VMAT plan compliance, the tool demonstrated balanced performance, with sensitivity, specificity, positive predictive value, negative predictive value, and accuracy at 83.33%, 83.33%, 76.92%, 88.24%, and 83.33%, respectively. In summary, our decision support tool showcased robust statistical performance in the validation group, affirming its reliability in real-world clinical applications.

## 4 Discussion

In this study, we have successfully developed a decision support tool rooted in pre-treatment CT image features and characteristics of OAR and PTV. The primary aim is to empower physicians in selecting the most suitable radiotherapy modality, whether VMAT or IMRT. Our decision support tool demonstrates significant advantages and practicality. Firstly, it identifies patients likely to benefit from VMAT at an early stage, enabling prompt adoption of effective radiation therapy techniques by physicians and providing patients with more individualized treatment options. Secondly, the tool exhibits high positive predictive values for plan compliance, delivering reliable information to physicians and patients and thereby reducing unnecessary workload associated with treatment planning and optimization.

Esophageal cancer, classified into upper, middle, and lower thoracic segments, presents distinctive characteristics that impact the optimal selection of radiotherapy techniques. Comparative studies by Zhang et al. and Lin et al. examined IMRT and VMAT plans, revealing superior CI for PTV with double-arc VMAT in upper thoracic esophageal cancer (16, 24). Our findings resonate with existing literature, underscoring the varied responses of esophageal cancer across anatomical locations to radiotherapy techniques (16, 25). Specifically, VMAT demonstrates significant superiority in conformity and dosimetry for upper thoracic esophageal cancer (24), while dosimetric differences between VMAT and IMRT are less pronounced in the middle and lower segments (16, 26). Furthermore, we observed variations in VMAT and IMRT’s efficacy in protecting OAR based on tumor location. Zhang et al. found significantly lower V20 and V30 for upper thoracic VMAT plans compared to IMRT (24), while Lin et al. reported lower average lung dose and V5 for IMRT in upper thoracic esophageal cancer, with lower values for middle and lower thoracic segments compared to VMAT (16). Consistent with most studies, our results indicate superior heart protection with VMAT over IMRT, albeit with location-specific differences. This further validates the rationale behind the design of our decision support tool, enhancing the precision of treatment technique selection based on individual patient conditions.

VMAT and IMRT radiotherapy techniques may present different characteristics in balancing target coverage and normal organ protection due to their inherent technical features. The diverse response of esophageal cancer at different locations to radiotherapy necessitates an analysis of factors influencing dosimetric differences and the identification of patient cohorts suitable for different technologies, emphasizing the importance of personalized treatment (27). Studies by Yang et al. suggest that VMAT is more suitable for small target areas, while IMRT is preferable for large target areas, with no significant differences for medium-sized target areas (28). In practical clinical scenarios, physicists often need to design two plans for the same patient to compare and choose the optimal approach. However, due to the significantly longer optimization time for VMAT plans compared to IMRT plans, predicting the likelihood of an advantageous plan through measurable parameters can effectively enhance clinical efficiency.

Our study demonstrates that predictive models P1 and P2, based on the total lung volume to PTV volume ratio (TLV/PTV ratio), effectively predict the compliance of IMRT and VMAT plans. Specifically, P1 ≥ 0.72 increases the likelihood of non-compliance with IMRT plans, while P2 ≥ 0.62 increases the likelihood of non-compliance with VMAT plans. When non-compliance is predicted, clinicians can adjust target delineation and reassess compliance, thereby saving substantial time in plan reproduction and optimization. Several studies support the importance of the TLV/PTV ratio in radiotherapy planning. Meltem et al. found that found that in helical tomotherapy for upper thoracic esophageal cancer, a TLV/PTV ratio ≥ 7 and bilateral lung volume ≥ 3500 cc can predict compliance with lung dose limits (29). Ueyama et al. identified the TLV/PTV ratio as a crucial factor influencing post-radiotherapy radiation pneumonitis (30). Gong et al. showed that controlling respiratory-adjusted lung volume significantly reduces lung dose (31). These findings corroborate our model and highlight the clinical value of the TLV/PTV ratio (32). Although individual PTV volume did not show statistical significance in our analysis, it remains a key parameter in radiotherapy planning. Our study aims to construct a comprehensive predictive model that considers multiple factors beyond statistical significance. By including PTV volume, we ensure our model captures all variables affecting treatment plan compliance, providing clinicians with a more holistic assessment tool. While our study focuses on optimizing IMRT and VMAT plans and developing a decision support tool, we did not directly assess long-term survival rates, quality of life, or treatment-related toxicity. However, optimized treatment plans are expected to positively influence these outcomes indirectly. High-quality radiotherapy plans have been shown to improve local control and reduce toxicity. Future research will aim to validate our decision support tool in larger clinical cohorts and evaluate its impact on these critical long-term outcomes.

The clinical utility of our decision support tool is a key highlight of this study. It simplifies the planning process by eliminating the need to design two separate plans for the same patient, thereby significantly improving workflow efficiency. However, several limitations should be acknowledged. Firstly, our study requires validation with larger-scale clinical data for further optimization. Secondly, the model currently uses a limited set of features. Future research should explore additional predictive factors, including biological and genetic characteristics, to enhance model accuracy. Thirdly, while we considered several key factors influencing radiotherapy plan selection, other important variables such as individual patient characteristics and treatment history were not included. Investigating these factors could provide a more comprehensive understanding of treatment planning decisions for thoracic esophageal cancer. Another limitation is the lack of direct comparison with automatic planning tools, such as those developed by the Pinnacle treatment planning system. While our tool streamlines the planning process and offers personalized recommendations, comparing it with automatic planning tools could reveal the relative strengths and weaknesses of each approach. Future studies should explore these comparisons to strengthen our findings and assess potential synergies between different planning methodologies. Despite these limitations, our results underscore the promising clinical applicability and innovation of our decision support tool. Although we did not evaluate long-term outcomes such as survival rates, quality of life, and treatment-related toxicity, the primary objective was to enhance the efficiency and accuracy of the planning process. We believe that these improvements will indirectly benefit long-term outcomes.

In conclusion, our study successfully introduces a decision support tool that leverages pre-treatment features to assist physicians in refining radiotherapy techniques for esophageal cancer patients. Beyond aiding in the VMAT or IMRT choice, the tool, driven by predictive models P1 and P2, pioneers an early assessment of plan compliance likelihood. This empowers clinicians to make informed adjustments, mitigating delays and inefficiencies associated with suboptimal plans. The decision support tool plays a pivotal role in streamlining decision-making, reducing workloads, and improving overall clinical efficiency. Looking forward, our focus remains on refining the model, expanding validation efforts, and optimizing performance to better serve clinical practice. Our study not only introduces a sophisticated tool but lays the foundation for a more patient-centric approach to esophageal cancer radiotherapy planning.

## Data availability statement

The original contributions presented in the study are included in the article/**Supplementary Material**. Further inquiries can be directed to the corresponding author.

## Ethics statement

The studies involving humans were approved by Medical Ethics Committee of Xi’an Jiaotong University. The studies were conducted in accordance with the local legislation and institutional requirements. Written informed consent for participation was not required from the participants or the participants’ legal guardians/next of kin in accordance with the national legislation and institutional requirements.

## Author contributions

SH: Conceptualization, Data curation, Formal analysis, Investigation, Methodology, Project administration, Resources, Software, Supervision, Validation, Visualization, Writing – original draft, Writing – review & editing. YL: Conceptualization, Data curation, Formal analysis, Investigation, Methodology, Project administration, Resources, Software, Supervision, Validation, Visualization, Writing – original draft, Writing – review & editing. YK: Data curation, Formal analysis, Funding acquisition, Investigation, Methodology, Software, Writing – original draft, Writing – review & editing. XH: Data curation, Methodology, Writing – original draft, Writing – review & editing. RZ: Data curation, Methodology, Writing – original draft, Writing – review & editing. WS: Data curation, Methodology, Writing – original draft, Writing – review & editing. HM: Methodology, Writing – original draft, Writing – review & editing. PH: Data curation, Resources, Writing – original draft, Writing – review & editing. XC: Data curation, Resources, Writing – original draft, Writing – review & editing.
