# Supplementary material for: Innovative regression model-based decision support tool for optimizing radiotherapy techniques in thoracic esophageal cancer
Source: Front Oncol. 2024 Jul 24;14:1370293. doi: 10.3389/fonc.2024.1370293 (PMC11303316; doi:10.3389/fonc.2024.1370293)
Supplement: Supplementary file 1 [file DataSheet_1.pdf]

Supplementary Tables

Supplementary Table S1 Detailed Prediction Score for Each Model

Model	Sensitivity	Specificity	FPR	FNR	Accuracy	MCC	AUROC(95% CI)
P1 Model	71.43%	84.62%	15.38%	28.57%	80.00%	0.560	0.901(0.754-1.000)
P2 Model	84.62%	85.71%	14.29%	15.38%	85.00%	0.684	0.912(0.783-1.000)

FPR, false positive rate; FNR, false negative rate; MCC, Matthews correlation coefficient; AUROC, area under the receiver operating characteristic curve.

Supplementary Figures

Supplementary Figure S1. Comparison of IMRT and VMAT Plans for Three Different Representative Patients. In the DVH curves, the IMRT plans are represented by the square-shaped continuous curve, while the VMAT plans are depicted by the triangle-shaped continuous curve.

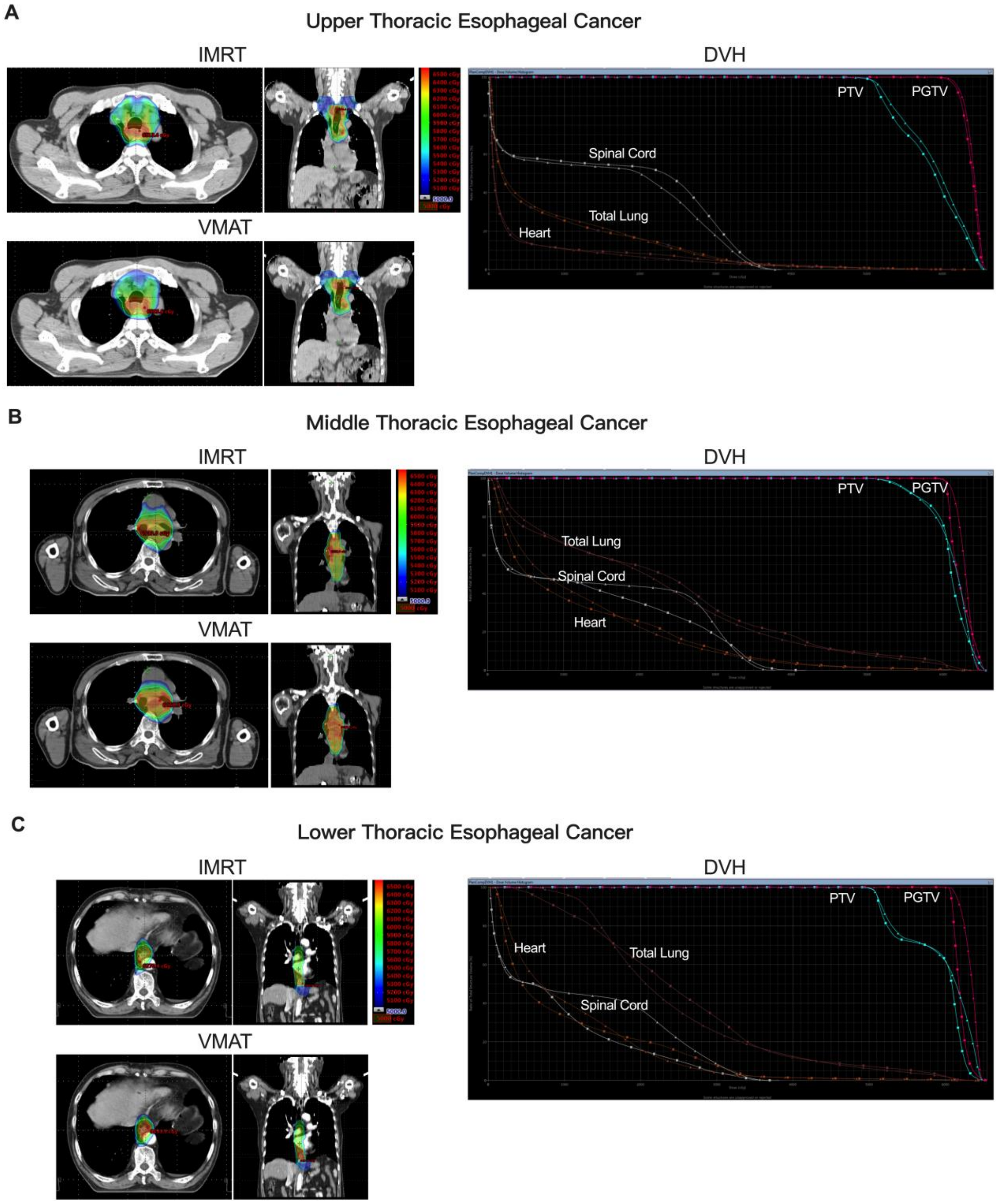
A
Upper Thoracic Esophageal Cancer
IMRT
VMAT
DVH
PTV
PGTV
Spinal Cord
Total Lung
Heart
B
Middle Thoracic Esophageal Cancer
IMRT
VMAT
DVH
PTV
PGTV
Total Lung
Spinal Cord
Heart
C
Lower Thoracic Esophageal Cancer
IMRT
VMAT
DVH
PTV
PGTV
Heart
Total Lung
Spinal Cord
